# Sustaining the Merry Space farmer with pick-and-eat crop production

**DOI:** 10.1038/s41526-025-00513-9

**Published:** 2025-10-27

**Authors:** Lauren Blackwell Landon, Sydney R. Begerowski, Peter G. Roma, Sara E. Whiting, Suzanne T. Bell, Gioia D. Massa

**Affiliations:** 1 Behavioral Health & Performance Laboratory, Biomedical Research and Environmental Sciences Division, Human Health and Performance Directorate, KBR, at NASA Johnson Space Center, Houston, TX USA; 2 Behavioral Health & Performance Laboratory, Biomedical Research and Environmental Sciences Division, Human Health and Performance Directorate, NASA Johnson Space Center, Houston, TX USA; 3 NASA Exploration Research and Technology, NASA Kennedy Space Center, Kennedy Space Center, Merritt Island, FL USA

**Keywords:** Psychology, Psychology

## Abstract

Astronauts on long-duration space missions may benefit nutritionally and psychologically from growing and consuming fresh fruits and vegetables. Gardening and exposure to nature can improve mood, reduce stress, provide meaningful and enjoyable tasks, and provide sensory stimulation. We investigated the behavioral health benefits of farming in space. Twenty-seven long-duration astronauts on the International Space Station engaged in crop growth experiments and answered surveys about their experiences, reactions to farming, and consumption of fresh fruits and vegetables throughout their missions. Findings indicate generally positive responses such that astronauts found the crop growth tasks enjoyable, engaging, meaningful, and stimulating. Ratings of behavioral health outcomes were consistent over time, while perceived sensory stimulation enjoyment increased over time. Positive effects were stronger when astronauts engaged in the most enjoyable tasks (i.e., consuming and voluntary viewing of plants). We discuss the implications of farming in space as a resilience countermeasure in austere environments.

Humanity is inextricably tied to Earth’s nature. Nature provides sustenance, sensory stimulation, and psychological benefits. However, as humanity leaves Earth, access to nature diminishes significantly. Exploring how astronauts might carry nature and those benefits with them is important for the next phase of space exploration beyond Earth’s orbit. The increased distance and duration required for exploration missions to Mars present greater physical and psychological challenges to future spaceflight crews. Resupply and crew changes are not anticipated for such missions, thus requiring a single crew to carry all the necessary resources to live and operate independently of Earth. Communications with Earth will have limited bandwidth, blackout periods and intermittent losses of signal, and will be delayed up to 22 min one-way for Mars missions^1^. Comm delays and isolation can reduce social support from, and connectedness with, mission support personnel and family and friends^2–4^. In addition to the social and physical isolation and inherent danger of spaceflight, the austere environment may also include a lack of sensory stimulation (e.g., monotony in diet, daily tasks, and other sensory experiences). Similar conditions occur to varying degrees in spaceflight mission simulations^5,6^ and in naturally occurring analogs such as Antarctic stations^7^. Decrements in mood, morale, and interpersonal functioning have been noted in low-Earth orbit spaceflights to the International Space Station (ISS)^8,9^. However, such risks are currently well-mitigated by a suite of countermeasures, including pre-mission expeditionary skills training, real time communications with work and personal connections on Earth, resupply and crew care packages, exercise capabilities, crew rotations, task variety, views of Earth, and a varied dietary menu^10,11^. Planetary exploration missions will require a similar multi-pronged mitigation approach, but countermeasures will need to be effective without resupply or real-time communication with a distant Earth. The purpose of our study is to examine interacting with plants (e.g., tending to, consuming) as a behavioral health and performance countermeasure for astronauts on long-duration space missions. While there exist many anecdotal reports related to the psychological benefits of space farming and exposure to plants and fresh food in space, this is the first quantitative examination of the topic in long-duration spaceflight.

Crops may have many roles in future long duration space exploration. In longer term scenarios these can be the basis for bioregenerative life support, with crops able to generate a large percentage of the diet, and with plants capturing carbon dioxide and generating oxygen through photosynthesis^12^. The importance of these roles will scale with increased crop growth. However, prior to bioregenerative crop production, cultivating edible plants could be a critical component of an Earth-independent countermeasure suite for mitigating the psychological and physical stresses of exploration spaceflight and surface habitation. The primary purpose of a near-term space crop production system is twofold: (1) provide a renewable source of fresh vitamins and other nutrients, and (2) provide menu variety to avoid menu fatigue and maintain an appropriate appetitive drive to prevent excessive body mass loss^13,14^. Larger-scale crop growth systems have been successfully deployed for these purposes at several Antarctic stations during the winter-over season when stations are inaccessible for resupply^15,16^.

There are also secondary benefits of spaceflight crop growth related to behavioral health and performance. The Biophilia Hypothesis states that humans are predisposed with an innate affinity for connection with nature and that exposure to nature has psychological and physiological benefits^17^. A meta-analysis examining this hypothesis has demonstrated varied benefits from exposure to plants, gardening, and natural environments including an increase in positive emotions^18^. Other studies support the positive connection between neighborhood green space and mental health^19^. Horticultural therapy, while peripheral to more traditional clinic-based approaches, has demonstrated benefits to mood, stress reduction, weight management, and interpersonal connectedness^20^. When considering indoor plants specifically, the presence of plants indoors is associated with cognitive performance benefits and health benefits in the form of lower blood pressure, shorter hospitalizations, reduced use of analgesics and lower ratings of pain, reduced anxiety and fatigue, and more positive feelings and higher satisfaction about their rooms^21,22^. Generally, gardening and exposure to nature has been shown to be therapeutic in terrestrial settings with reductions in anxiety and negative mood, and improvements in stress levels and positive mood, and it is likely these positive effects translate to extreme environments like spaceflight^23^.

Caring for plants and eating fresh food have been shown to provide psychological benefits for austere environments such as Antarctica^24,25^. Antarctic crewmembers describe psychological benefits from the diverse sensory stimuli (e.g., relishing the sights, smells, textures, and/or change in humidity and temperature) the station “greenhouses” afford compared to their monotonous and austere surroundings^26–28^. Astronauts anecdotally report morale and stress reduction benefits of eating fresh foods and tending to plants grown in small-scale aboard the Russian Mir station and International Space Station^8,25,29,30^. In the ISS astronaut journals study^8,30^, food was one of the top 10 items discussed by the astronauts who participated. The study principal investigator, Jack Stuster, suggested “Food assumes added importance when access to friends, family, leisure pursuits and other normal sources of gratification are denied. The importance of food during isolation and confinement is well-known to the managers of oil rigs, commercial ships, Antarctic research stations and nuclear submarines, all of whom serve large quantities and varieties of high-quality food daily”^30^. The availability of fresh food may enhance the desire and consumption of food in general, which is important for maintaining crew health.

Cultivating edible crops in space is a complex process that requires substantial workload for successful yields. Antarctic crews tending greenhouse crops have spent approximately 23 crewmember-hours per week on average caring for the plants and growth systems^27,31^. However, crew time and workload can vary by system design, especially by the degree of automation. The additional time and workload of plant growth must be designed in balance with other mission tasks and health requirements, such as maintenance and systems monitoring, exercise, onboard science, public outreach, and duty day limits. Astronauts are also encouraged to engage in activities to mitigate stress and support wellbeing. While these activities may not be scheduled, they can add to the overall time burden. However, engaging, enjoyable, and meaningful work activities may serve a dual purpose by accomplishing mission objectives, e.g., the task of crop growth, while also supporting behavioral health, e.g., via plant interactions. The degree of benefit from tending and viewing plants is also likely to depend on individual differences, such as life experiences with nature and plant care, and personal interests in related hobbies or fields of study. Meaningful work can act as a resilience countermeasure in work and extreme environments, supporting positive mood and mitigating negative mood and stress^32,33^. Meaningfulness of crop growth tasks may be related to personal preference for tending to living things and watching plants grow, and may also stem from producing fresh, nutritious food for the crew to enhance their diet with a greater variety of pleasing food.

Exposure to plants in a habitat largely devoid of Earth’s nature may fulfill biophilic needs to some extent, maintaining a connection to Earth and engendering positive mood, wellbeing, and connections to others. Conversely, a strong biophilic-like connection with the plants could potentially trigger decrements in morale and mood if the crops fail. Smaller-scale plant growth experiments in NASA’s Veggie and Advanced Plant Habitat growth chambers aboard the ISS encountered growth failures and plant death, which have led to insufficient yield to conduct sensory evaluation tasting sessions, a highly anticipated activity for most crews. One participant in this study stated that “having fresh salad really made my week!” Disappointment and stress from a shortage of preferred, fresh foods or perceived poor task performance in growing crops may negate or weaken the psychological benefits of attempting to grow the plants.

The Vegetable Production System (Veggie) has been in operation on ISS since 2014 (with a second unit installed in 2017) and the Advanced Plant Habitat (PH or APH) crop growth chamber was installed in 2017 and first operated in 2018^34,35^. The primary goals for these hardware systems were to conduct plant science research in space, and components of this science included approaches to optimize plant growth and evaluate crop cultivars and cultivation practices, microbial food safety, and sensory acceptability. NASA plant science researchers and food and nutrition researchers partnered with behavioral health researchers to consider the behavioral health and performance implications associated with conducting crop growth studies in spaceflight.

The objective of our study was to examine behavioral health outcomes associated with tending to plants and consuming crops grown during long-duration spaceflight missions. This is the first study to systematically and quantitatively assess this phenomenon with spaceflight crews. We explored which aspects of horticulture are most salient and psychologically beneficial to astronaut health and wellbeing. We hypothesized that engaging with plants during a mission will have a beneficial effect as evidenced by reported positive behavioral health outcomes such as enjoyment, positive mood and enhanced wellbeing, connection to Earth, connections to others, and performance. We also hypothesized that interacting with plants will be a source of positive sensory stimulation for the crew. Pre-mission predictors, post-mission outcomes, and reported time spent engaging with the plants as well as in-mission changes over time were also considered.

## Results

All measures for pre-mission, in-mission, and post-mission were processed, visualized, and inspected for quality. Outliers were assessed by evaluating the interquartile range and running extreme studentized deviate tests for in-mission reported time spent on specific tasks. We identified outliers for set up (*N* = 1), watering (*N* = 3), pollinating (*N* = 1), thinning (*N* = 1), debris removal (*N* = 1), photography (*N* = 5), voluntary viewing (*N* = 3), and consuming (*N* = 1). These outliers tended to occur in the early stages of the project, reflecting initial set up and troubleshooting efforts. Accordingly, because these instances were not reflective of nominal operations once the crop growth chamber hardware was fully installed, the data was removed prior to analyses. Crewmembers averaged 6.17 (SD = 4.85) h spent on crop growth system tasks per month while time spent of specific tasks varied (Table 1).

**Table 1:** Summary of monthly minutes spent on crop growth tasks

Task	*N*	*M*	SD	Min	Max
Set up	22	74.77	64.11	5	200
Harvesting	21	56.67	51.53	5	200
Watering	39	43.21	34.19	5	120
Clean up	22	38.18	31.42	10	120
Plant thinning	13	28.85	19.49	5	60
Debris removal	15	20.33	15.86	5	60
Consuming	24	20.08	9.72	2	40
Wick opening	12	16.67	9.61	5	30
Voluntary viewing	34	15.50	7.90	2	30
Photography	49	14.22	9.48	2	40
Pollinating	10	6.50	2.42	5	10

*N* Number of data collection instances, *M* Mean, *SD*Standard Deviation, *Min* Minimum value, *Max* Maximum value.

### Descriptive analyses

For pre-mission plant interactions, crewmembers appeared to have some familiarity with the work required to grow plants. Familiarity of tending to plants as an adult was moderate (*M* = 4.27, SD = 1.54). Enjoyment related to tending to plants was moderately high (*M* = 5.96, SD = 1.11), as was engagement (*M* = 5.65, SD = 1.09) and meaningfulness (*M* = 6.00, SD = 1.02). Crewmembers generally rated interacting with plants to be a strong source of sensory stimulation for sight (*M* = 6.27, SD = 1.00), touch (*M* = 5.72, SD = 1.17), smell (*M* = 6.23, SD = 0.86), and taste (*M* = 6.31, SD = 0.97).

**Fig. 1:**
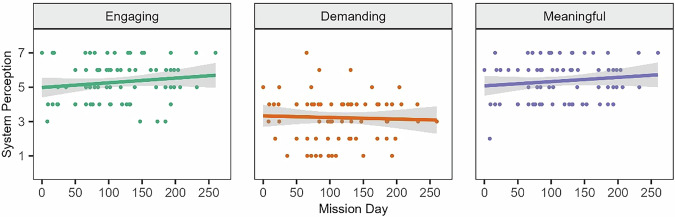
Perceptions of crop growth system over time. Higher scores indicated a greater degree of that construct. Colored dots represent scores from each survey administration. Colored lines illustrate linear trends and gray bars reflect a 95% confidence interval.

To assess perceptions of the crop growth system, crewmembers rated the extent to which working with the system was engaging, demanding, and meaningful. Overall, the system was rated slightly engaging (*M* = 5.29, SD = 1.21), slightly easy (*M* = 3.22, SD = 1.33), and slightly meaningful (*M* = 5.36, SD = 1.21). Ratings were generally consistent throughout the mission, with no significant changes in crop growth system perceptions over time (Fig. 1).

Acts of consuming and voluntary viewing appeared to be the most enjoyable for the crew (Table 2). Average rankings for these tasks were near scale maximum and minimum scores did not drop below the scale midpoint. Harvesting, plant thinning, watering, and wick opening also averaged high. Set up, photography, pollinating, and debris removal averaged near the midpoint, suggesting crews neither enjoyed nor disliked these tasks. Finally, clean up duties appeared to be slightly unpleasant for crew with an average rating of 3.50. Clean up could refer to sanitizing the produce with sanitizing wipes prior to consumption, or cleaning up the hardware after the growth study was complete. Enjoyment ratings appeared to be generally consistent. Repeated measures correlations between task enjoyment and time revealed no significant relationships.

**Table 2:** Summary of crop growth task enjoyment

Task^a^	*N*	*M*	SD	Min	Max
Consuming	24	6.25	1.15	4	7
Voluntary viewing	34	6.00	1.10	4	7
Harvesting	21	5.52	1.63	2	7
Plant thinning	13	5.23	1.01	4	7
Watering	39	5.15	1.09	3	7
Wick opening	12	5.00	1.04	4	7
Set up	22	4.86	1.42	1	7
Photography	49	4.71	1.19	2	7
Pollinating	10	4.70	0.95	3	6
Debris removal	15	4.40	1.12	3	6
Clean up	22	3.50	1.14	1	5

*N* Number of data collection instances, *M* Mean, *SD* Standard Deviation, *Min* Minimum value, *Max* Maximum value.

^a^Crewmembers ranked tasks from 1 = very unpleasant to 7 = very enjoyable.

The perceived behavioral health and performance effects of the crop growth experiment ranged from moderate to high. For Earth connection and desires to consume and harvest, the averaged perceived impacts of the crop growth system were slightly above the scale midpoint of 4, suggesting the crop growth system facilitated these outcomes to some extent. Other outcomes averaged near the scale midpoint (Table 3).

**Table 3:** Perceived effects of interacting with the crop growth system

Outcome^a^	*N*	*M*	SD	Min	Max
Mood	76	5.25	1.21	3	7
Performance on Mission Tasks	76	4.79	1.00	3	7
Wellbeing	76	5.22	1.23	2	7
Relationship with Crewmembers	76	5.04	1.16	3	7
Earth Connection	77	5.42	1.22	2	7
Desire to Harvest Plants	76	5.37	1.30	2	7
Desire to Consume Plants	76	5.42	1.38	1	7
Desire to Consume Food	76	4.55	1.00	3	7

*N* Number of data collection instances, *M* Mean, *SD* Standard Deviation, *Min* Minimum value, *Max* Maximum value.

^a^Crewmembers rated how interacting with the crop growth system impacted the above outcomes from 1 = diminished to 7 = enhanced.

**Fig. 2:**
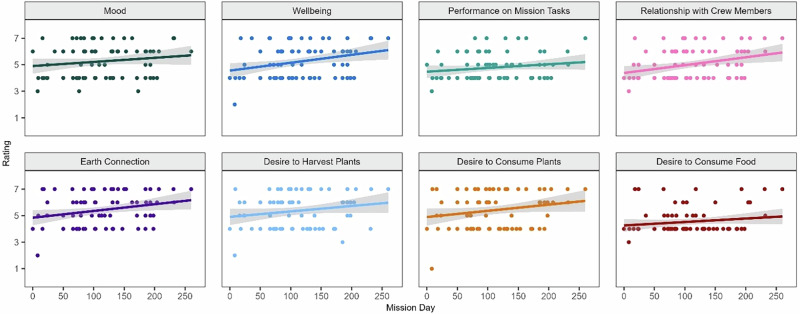
Perceived effects of interacting with the crop growth system over time. Higher scores indicated a greater degree of that construct. Colored dots represent scores from each survey administration. Colored lines illustrate linear trends and gray bars reflect a 95% confidence interval.

Perceived effects of interacting with the crop growth system were also assessed over time (Fig. 2). Repeated measures correlations revealed small positive correlations with time for performance of mission tasks (*r* = 0.31, *p* = 0.028), relationships with crewmembers (*r* = 0.35, *p* = 0.012), and desire to consume food (*r* = 0.34, *p* = 0.014). All other perceived effects were stable across time.

**Fig. 3:**
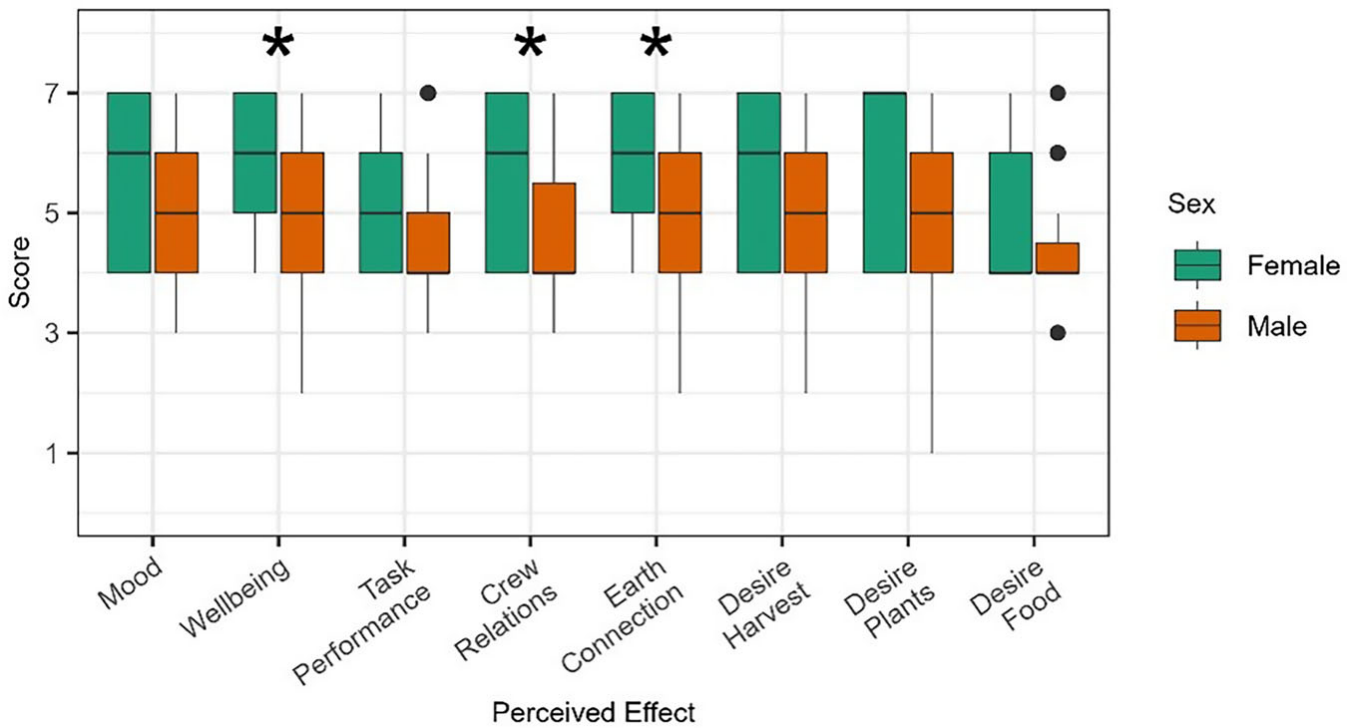
Perceived effects by sex. *p < 0.05. Black dots represent outliers. Significant differences were reported across sex for wellbeing, relationship with other crew members, and Earth Connection.

Finally, perceived effects were evaluated for differences by sex of the crewmember (Fig. 3). Perceived benefits differed by sex for wellbeing (*b* = −0.94, *t* = −2.38, *p* = 0.03), relationship with crewmembers (*b* = −0.93, *t* = −2.59, *p* = 0.02), and Earth connection (*b* = −0.88, *t* = −2.19, *p* = 0.04). Across these, female astronauts reported higher ratings than male astronauts.

The perceived sensory stimulation effects of the crop growth experiment varied across the scale, with the average perception of sight and smell slightly above the midpoint (Table 4). Perceptions of touch and taste neared the midpoint.

**Table 4:** Perceived sensory effects of the crop growth system

Outcome^a^	*N*	*M*	SD	Min	Max
Sight	97	5.40	1.46	1	7
Touch	97	4.86	1.31	1	7
Smell	97	5.31	1.44	2	7
Taste	96	5.10	1.40	3	7

*N* Number of data collection instances, *M* Mean, *SD* Standard Deviation, *Min* Minimum value, *Max* Maximum value.

^a^Crewmembers rated the crop growth system as a source of sensory stimulation from 1 = Very Unpleasant to 7 = Very Pleasant.

**Fig. 4:**
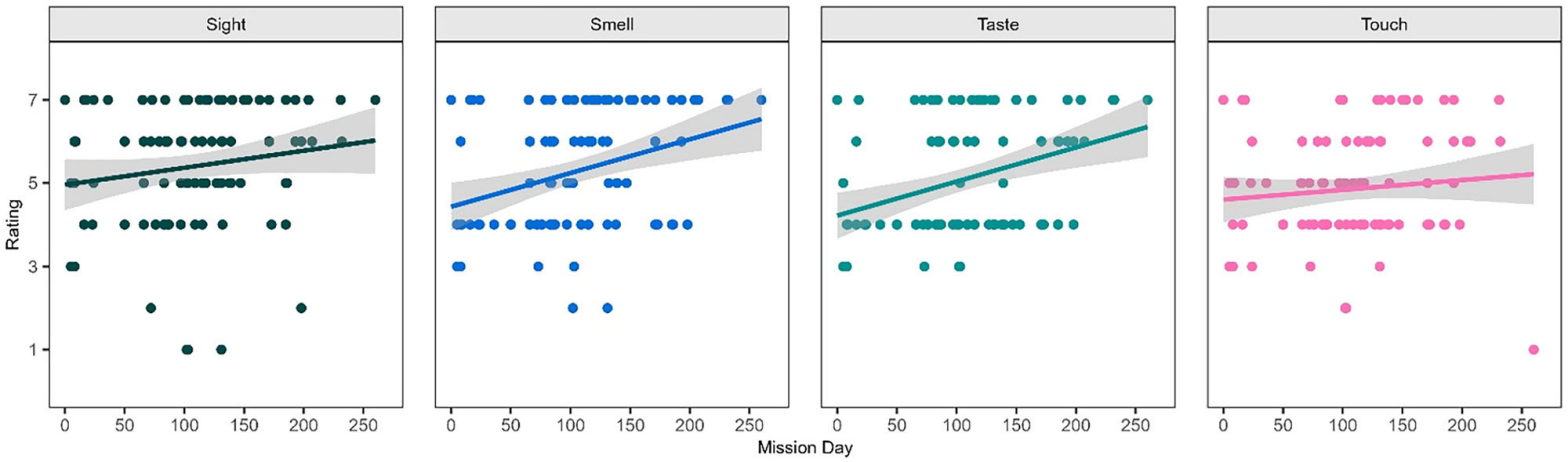
Perceived sensory effects of crop growth system over time. Higher scores indicated a greater degree of that construct. Colored dots represent scores from each survey administration. Colored lines illustrate linear trends and gray bars reflect a 95% confidence interval.

Perceived sensory effects of the crop growth system were also correlated over time (Fig. 4). Repeated measures correlations revealed a small correlation between time and sight (*r* = 0.03, *p* = 0.011). Stronger correlations were detected for smell (*r* = 0.40, *p* = 0.000), taste (*r* = 0.33, *p* = 0.005), and touch (*r* = 0.26, *p* = 0.023). The sensory effects of interacting with the crop growth system was increasingly pleasant over time. Finally, perceived sensory effects were tested for differences across sex, with no significant differences found.

### Data analysis

Our hypotheses suggested that, first, engaging with plants and the crop growth system would have a beneficial effect on behavioral health outcomes, and second, that interacting with plants would be a source of sensory stimulation for the crew. We first tested this with a series of linear mixed model analyses with random subject intercepts to assess various factors of crop growth system (e.g., task types, interaction levels) and behavioral health outcomes. Two controls were assessed in each model: previous experience with plants and pre-mission levels of enjoyment related to tending to plants. Residual normality was assessed using QQ plots and the Shapiro-Wilk test. Robust linear mixed models were conducted using R package *robustlmm* when assumptions were violated^36^. All analyses were completed in RStudio using R 4.4.2.

A suite of models was run to analyze perceived impacts when engaging with the crop growth system. The False Discovery Rate method was applied to adjust for each multiple comparison. When appropriate, pairwise comparisons were assessed using Tukey’s HSD to adjust for Type-1 error. All results reflect adjusted *p*-values.

**Fig. 5:**
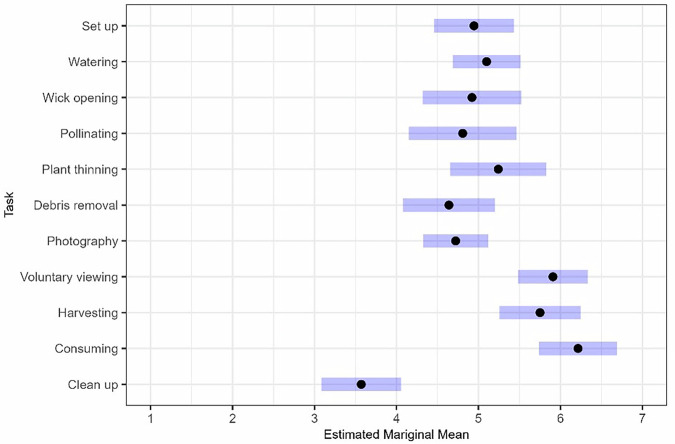
Enjoyment across tasks. Black dots represent estimated mariginal means for enjoyment ratings. Purple bars reflect a 95% confidence interval.

Task enjoyment differed across task type (*b* = 3.57, adjusted *p* < 0.001). Previous pre-mission plant experience and pre-mission plant enjoyment levels were included as control variables; however, no significant effects were found. Pairwise comparisons on a trimmed model, which removed the nonsignificant control variables, revealed statistically significant differences between some tasks on enjoyment. A summary of estimated marginal means is depicted in Fig. 5. Generally, tasks were rated moderately to moderately high on the enjoyment scale, providing support for our first hypothesis.

Clean up was rated significantly less enjoyable than all other tasks: consuming (*b* = −2.64*, p*_adjusted_ < 0.000), harvesting (*b* = −2.18*, p*_adjusted_ < 0.000), voluntary viewing (*b* = −2.34*, p*_adjusted_ < 0.000), photography (*b* = −1.15*,* adjusted *p* < 0.000), debris removal (*b* = −1.07*, p*_adjusted_ < 0.025), plant thinning (*b* = −1.67*, p*_adjusted_ < 0.001), pollinating (*b* = −1.24*, p*_adjusted_ = 0.023), wick opening (*b* = −1.35*, p*_adjusted_ = 0.002), watering (*b* = −1.53*, p*_adjusted_ < 0.001), and set up (*b* = −1.37*, p*_adjusted_ < 0.001).

Consuming was rated significantly higher than all tasks except plant thinning, harvesting, and voluntary viewing. Specifically, consuming was rated more enjoyable than photography (*b* = 1.49*, p*_adjusted_ < 0.000), debris removal (*b* = 1.57*, p*_adjusted_ < 0.000), pollinating (*b* = 1.41*, p*_adjusted_ = 0.004), wick opening (*b* = 1.29*, p*_adjusted_ = 0.004), watering (*b* = 1.12*, p*_adjusted_ < 0.000), and set up (*b* = 1.27*, p*_adjusted_ < 0.000).

Similarly, voluntary viewing was rated as significantly more enjoyable than photography (*b* = 1.87*, p*_adjusted_ < 0.000), debris removal (*b* = 1.27*, p*_adjusted_ = 0.001), watering (*b* = 0.09*, p*_adjusted_ = 0.009), and set up (*b* = 0.96*, p*_adjusted_ = 0.008) but not for plant thinning, pollinating, or wick opening. Finally, harvesting was rated more enjoyable than photography (*b* = 1.03*, p*_adjusted_ = 0.001) and debris removal (*b* = 1.11*, p*_adjusted_ = 0.019). All other comparisons between tasks on enjoyment were nonsignificant.

**Fig. 6:**
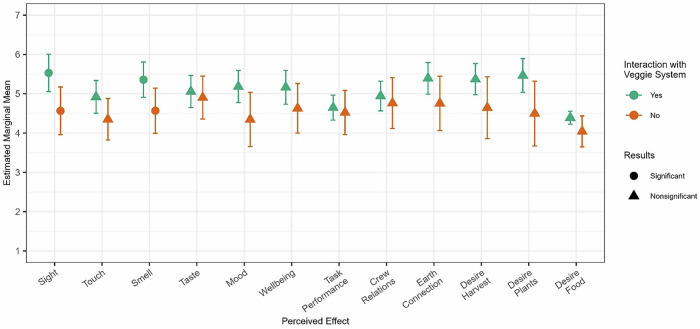
Summary of perceived effects when interaction with crop growth system. Error bars reflect a 95% confidence interval. Significance is determined by adjusted *p* values. Significant relationships are denoted by circles and reflect a difference in means between those who engaged in the sub task vs. those who did not for the given outcome.

Perceived effects of interacting with the crop growth system, including behavioral outcomes and sensory stimulation, were assessed for differences based on crop growth involvement. We found support for our second hypothesis regarding sensory stimulation. Interacting with the crop growth system was linked to perceptions of sight (*b* = 0.97*, p* = 0.001, *p*_adjusted_ = 0.012), with those completing at least one crop growth task in the last 30 days reporting more enjoyable visual perceptions (*M* = 5.53, *SE* = 0.23) than those with no interaction (*M* = 4.56, *SE* = 0.30). Interaction was also related to smell (*b* = 0.79*, p* = 0.005, *p*_adjusted_ = 0.030), with those who interacted reporting more enjoyable olfactory perceptions (*M* = 5.36, SE = 0.21) than no interaction (*M* = 4.57, SE = 0.29). However, this analysis did not provide support for our first hypothesis. Impacts of interaction on mood (*b* = 0.84*, p* = 0.014, *p*_adjusted_ = 0.056), touch (*b* = 0.57*, p* = 0.025, *p*_adjusted_ = 0.060), and desire to consume plants (*b* = 0.97*, p* = 0.022, *p*_adjusted_ = 0.060) were nonsignificant. Group differences are depicted in Fig. 6. No significant differences were detected for taste, wellbeing, performance on mission tasks, relationship with crewmembers, Earth connection, desire to harvest plants, or desire to consume food. For models that were significant, previous adult experience with plants and baseline levels of enjoyment in tending to plants were added as control variables, but were not significant.

### Comparing most and least enjoyable tasks

Earlier analyses suggested considerable differences in enjoyment across tasks (Fig. 5). Accordingly, perceived effects were also evaluated based on completing the most and least enjoyable tasks. Clean up was designated as least enjoyable, as it was rated significantly lower (*p* < 0.05) than all other tasks. Voluntary viewing and consuming were classified as the most enjoyable tasks. These tasks received the highest average enjoyment ratings and were never rated below the scale midpoint. Additionally, ratings of enjoyment between these tasks were nonsignificant (*b* = 0.306, *p*_adjusted_ = 0.976). Crewmembers’ perceived effects were assessed based on whether they engaged in cleaning up over the past 30 days. For most enjoyable, outcomes were assessed as to whether they engaged in voluntary viewing, consuming, or both. Table 5 summarizes these results.

**Table 5:** Estimated Marginal Means across Most and Least Enjoyable Tasks

	Most Enjoyable^a^					Least Enjoyable^b^				
		Interaction		No Interaction			Interaction		No Interaction	
Outcome	*b*	*M*	SE	*M*	SE	*b*	*M*	SE	*M*	SE
*Sensory*^c^										
Sight	1.03**	5.82	0.26	4.79	0.27	−0.01	5.53	0.23	4.57	0.30
Touch	0.73**	5.09	0.22	4.35	0.23	−0.35	4.92	0.21	4.35	0.27
Smell	0.92**	5.85	0.25	4.92	0.26	0.56	5.36	0.22	4.57	0.29
Taste	1.06**	5.63	0.21	4.57	0.22	0.09	5.06	0.20	4.90	0.27
*Behavioral Health*^d^										
Mood	1.10**	5.54	0.19	4.44	0.21	−0.23	5.18	0.20	4.34	0.34
Wellbeing	1.78**	5.53	0.19	4.58	0.21	−0.02	5.16	0.22	4.63	0.32
Performance on Mission Tasks	0.43*	4.87	0.16	4.44	0.18	0.19	4.65	0.16	4.52	0.28
Relationship with Crewmembers	0.45*	5.19	0.19	4.73	0.21	0.30	4.94	0.18	4.76	0.32
Earth Connection	0.67**	5.59	0.21	4.91	0.22	−0.14	5.39	0.20	4.76	0.34
Desire to Harvest Plants	0.64*	5.59	0.22	4.95	0.25	0.07	5.37	0.19	4.64	0.39
Desire to Consume Plants	0.71**	5.62	0.23	4.91	0.25	−0.22	5.47	0.21	4.50	0.41
Desire to Consume Food	0.32	4.65	0.11	4.33	0.13	0.52**	4.39	0.08	4.04	0.20

*b* unstandardized coefficient, *M* Estimated Marginal Mean, *SE* Standard Error.

*p < 0.05, **p_adjusted_ < 0.05.

^a^Interaction for most enjoyable refers to crewmembers who voluntarily viewed and/or consumed plants in the past 30 days. No interaction can reflect completion of other tasks, or no interaction with the crop growth system.

^b^Interaction for least enjoyable refers to crewmembers who cleaned up in the past 30 days. No interaction can reflect completion of other tasks, or no interaction with the crop growth system.

^c^Crewmembers rated how the crop growth system affected their senses from 1 = Very Unpleasant to 7 = Very Pleasant.

^d^Crewmembers rated how the crop growth system impacted the above outcomes from 1 = Diminished to 7 = Enhanced.

We found some support for our first hypothesis, and consistent report for our second hypothesis when comparing the most versus least enjoyable tasks. Completion of clean up tasks was associated with desire to consume food in general (*b* = 0.52*, p* = 0.006, *p*_adjusted_ = 0.016). Specifically, crewmembers who engaged in clean up tasks reported more desire to consume food (*M* = 4.39, *SE* = 0.08) than those that did not (*M* = 4.04, *SE* = 0.20). There were no other relationships detected between clean up duties and perceived effects.

**Fig. 7:**
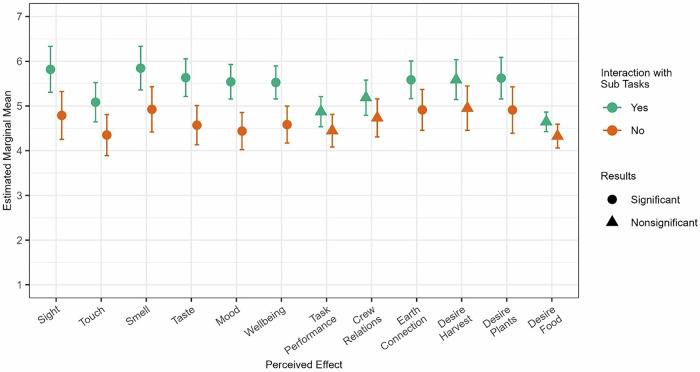
Summary of perceived effects when interacting with most enjoyable subtasks. Interaction with subtasks refers to crewmembers reported consuming and/or voluntary viewing. Error bars reflect a 95% confidence interval. Significance is determined by adjusted *p*-values. Significant relationships are denoted by circles and reflect a difference in means between those who engaged in the sub task vs. those who did not for the given outcome.

Crewmembers who voluntarily viewed and/or consumed plants reported higher perceived benefits across several areas (Fig. 7). Completion of these tasks was associated with more pleasant stimulation in the visual (*b* = 1.03*, p* = 0.000, *p*_adjusted_ = 0.002), olfactory (*b* = 0.92*, p* = 0.001, *p*_adjusted_ = 0.004), gustatory (*b* = 1.06*, p* = 0.000, *p*_adjusted_ = 0.000), and tactile (*b* = 0.73*, p* = 0.002, *p*_adjusted_ = 0.007) domains. Completing the two most enjoyable tasks also resulted in crewmembers indicating that working with the crop growth system enhanced mood (*b* = 1.10*, p* = 0.000, *p*_adjusted_ = 0.000), wellbeing (*b* = 1.78*, p* = 0.001*, p*_adjusted_ = 0.005), and their desire to consume plants (*b* = 0.71*, p* = 0.019, *p*_adjusted_ = 0.048). Finally, those who viewed the system and consumed were marginally more likely to perceive working with the crop growth system as a method to enhance task performance (*b* = 0.43*, p* = 0.025, *p*_adjusted_ = 0.058), their connection to Earth (*b* = 0.67*, p* = 0.004, *p*_adjusted_ = 0.013), crew relations (*b* = 0.45*, p* = 0.033, *p*_adjusted_ = 0.066), and desire to harvest (*b* = 0.64*, p* = 0.027, *p*_adjusted_ = 0.058). There were no detected effects for desire to consume food in general.

**Fig. 8:**
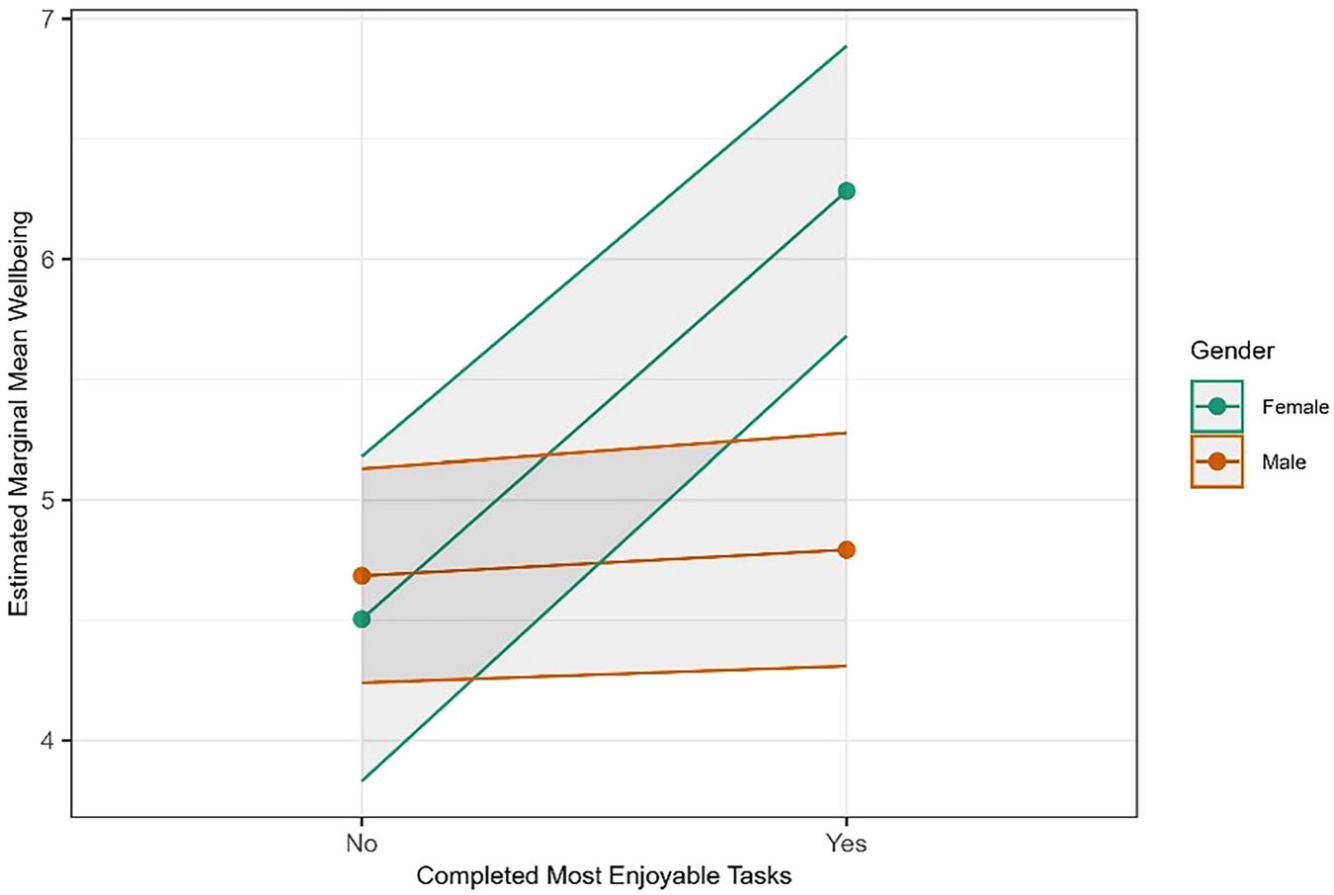
Relationship between completing most enjoyable tasks and wellbeing by sex. Interaction with subtasks refers to crewmembers reported consuming and/or voluntary viewing. Error bars reflect a 95% confidence interval.

Sex was examined as a potential control and was found to significantly interact with wellbeing. This outcome was unique such that completing the most enjoyable tasks predicted wellbeing, but this was further influenced by an interaction between most enjoyable task completion and sex (*b* = 1.67*, p* = 0.002, *p*_adjusted_ = 0.007). Specifically, female crewmembers who viewed the habitat and/or consumed produce indicated the system enhanced wellbeing (*M* = 6.27, SE = 0.30) more than those who did not (*M* = 4.49, SE = 0.35). Male crewmembers who completed these tasks had similar ratings (*M* = 4.78, SE = 0.23) to male crewmembers who did not (*M* = 4.67, SE = 0.22) as well as female crewmembers who did not (Fig. 8). Sex was not a significant factor for the remaining outcomes.

### Summary of participant comments about struggling and/or dying plants

At the end of each in-mission survey, crewmembers were asked to describe any experiences with any struggling or dying plants in the crop growth studies. Twelve comments by six crewmembers were recorded in total, with six comments stating that the crewmember had no feelings or effect from the experience. Two comments took the opportunity to focus on the crops or growing techniques that went well such as removing some struggling plants to help others grow well. Four comments recorded more negative experiences such as “disappointing,” “wonder[ing] if I did something wrong,” “tough, but the ones that do well make up for that negative aspect,” “a sobering reminder of the fragility of life,” and comments related to not knowing what went wrong and desiring more feedback from ground experts.

Twelve crewmembers recorded comments about struggling or dying plants in their post-mission survey. Six comments were neutral or stated there was no effect, “it’s just another science project.” Six comments described negative experiences including “a sad reminder of the fragility of life,” “feeling sorry or unhappy,” “a bit of a bummer”. Some negative comments were focused on the disappointment in the experiment not being successful, while others expressed a desire to communicate more with ground experts to understand what the crewmembers may do to improve their care of the plants. One comment expressed some frustration with removing dying plants because “[growing the unsuccessful plants] was a lot of work considering we didn’t get to harvest anything from them.” Generally, reported reactions to struggling or dying plants were neutral or mild.

## Discussion

Much has been anecdotally reported about the behavioral health benefits of farming in space. One astronaut participant, when speaking with study principal investigator Dr. Gioia Massa from space, stated, “*from a behavioral standpoint, I love working inside of this habitat. So it was no trouble for me to get those extra readings because when you’re inside, even leaning back, you still smell Earth. And we don’t have those smells at all. It’s very sterile, very mechanical. So, to be able to lean my head in and smell those moist plants and life is hugely beneficial. Thanks for letting me work on this today. I loved it!*” This study addresses a lack of empirical research into these oft-reported benefits.

We found support for our hypotheses that interacting with a crop growth system would offer behavioral health benefits and sensory stimulation. Examining each subtask involved in the overall planting, tending, harvesting, and consuming of crops offers unique insights for system designers and behavioral health experts seeking to optimize functional crop growth activities to also serve as a resilience countermeasure. Crewmembers reported many positive effects when engaging in the most enjoyable tasks (voluntary viewing and consuming), indicating that visible and physically accessible crop growth for consumption can be consistently supportive over time. Viewing, however, was one of the tasks with the lowest reported time spent. Several of the post-mission crew comments expressed the desire to have more plants, more time with the plants (“I peek at the plants daily, but it is very enjoyable to spend extended time near them, observing and interacting”), and to “move beyond the experiment” to truly engage in operational farming. Multiple crewmembers reported that they show their plants off to friends and family and during public affairs events, helping to find a relatable connection with others back home. Notably, photography, which is generally a favorite pastime for many on the ISS, was rated somewhat lower on enjoyment with respect to plant growth activities specifically. Comments explained that photographing the plants was challenging in the small locker setup, and perhaps negated the positives that accompany more general viewing. Thus, a greater volume of plants yielding consumable crops and optimizing the visual accessibility of the plants are likely targets for enhancing behavioral health benefits in future crop growth systems.

There were almost no beneficial effects related to the least enjoyable task: clean up. Clean up was also reported as a task that took considerable crew time. Sanitizing produce with wipes can take a long time, depending on the type and volume of produce. System designers should consider minimizing time-consuming tasks that were rated as not enjoyable (e.g., by creating an easy-to-clean crop growth habitat or a faster way to sanitize produce) in favor of allowing the allotted crop growth interaction time to be concentrated on tasks with the strongest behavioral health benefits. These enjoyable tasks also boosted sensory stimulation. The pattern of enjoyable tasks is such that most of the tasks early and midway through the crop growth lifecycle are rated neutral to moderately beneficial, while some of the later tasks (harvesting and consuming) are more potent morale boosters. One of the final tasks– clean up – had a notable ratings decrease into the unenjoyable range, perhaps diminishing some of the positive effects of the other final tasks of harvesting and consuming. Therefore, when possible, it may be beneficial to stagger plant growth lifecycles to support continuous beneficial effects throughout a mission. This has the added benefit of providing a more continuous nutrition supplement. Similarly, viewing is a task that can be done intermittently with easily visible plants to offer a regular behavioral health boost throughout the growth cycle. There was also no decline in the reported enjoyment or beneficial effects over time for the different crop growth tasks, so having a steady rotation of crops growing throughout a mission confers continuous benefits. One crewmember stated in their post-mission comments, “Keep it going!! The crew will do as many of these as you set up.” Another potentially challenging event for behavioral health – experiencing struggling or dying plants – seemed to have mild negative effects or no effects. Several in-mission and post-mission comments mentioned the desire to understand more about the best techniques to care for the plants and the desire to work more collaboratively with ground experts. Thus, pre-mission or even in-mission “just-in-time” training on plant growth systems may further offer countermeasure effects, such as increasing interest and motivation, cognitive stimulation, task mastery, and connections with Earth.

While pre-mission perceptions of plants were positive, these pre-mission perceptions did not predict crewmembers’ enjoyment in mission. Crop growth experiments may also offer a benefit to those that do not typically tend to plants on Earth, suggesting it can be a countermeasure for even novice space farmers. These novices may desire an even greater collaboration with ground experts to be successful. However, sex may need to be considered when allotting coverage of crop growth activities given that female astronauts in our sample reported increased wellbeing while male astronauts did not, although the small number of female astronauts in the study limit firm conclusions. Crew comments mentioned onboard discussions about the best way to care for the plants and the shared excitement when plants started to grow. Harvesting and consuming is also an opportunity for a positive shared experience with other crewmembers. Optimizing the positives of crop growth and exposure to plants while reducing time spent on hardware and produce cleaning can provide a unique behavioral health countermeasure, while also providing nourishment.

These results are particularly encouraging given the small, experimental nature of the onboard crop growth system studied. It is possible that some statistical analyses were underpowered due to the small sample size, and continued research in this area may shed more light on the relationship between space farming and behavioral health. Future research should include similar aims and outcomes with larger crop growth systems and isolated operational settings, such as Antarctic field stations and other space analogs, to confirm and refine conclusions and recommendations for future crop growth system designs.

## Methods

### Participants

Participants were 27 long-duration ISS astronauts (19 males, 8 females) that interacted with the ISS plant system during crop growth experiments and consented to participating in the study, which was approved by the NASA Institutional Review Board (IRB Protocol 2457). These ISS crewmembers were assigned to standard- (~6 months) or extended- (~12 months) missions. For those in standard-duration missions (*N* = 23), crewmembers averaged 182 days (SD = 17.48) in flight, whereas those in extended-duration missions (*N* = 4) averaged 332 days (SD = 43.43). The number of data collection opportunities differed between participants due to differences in mission duration and specific crop growth experiment requirements.

### Measures

Twenty pre-mission demographic questions asked respondents to report their age, biological sex, education/profession, years of experience, analog/ICE/space experience, and teamwork experience. See survey in Supplementary Information.

The Behavioral Health & Performance (BHP) Veggie Survey has up to 34 items, which ask respondents to describe their experiences with plants and interacting with the Veggie or alternate plant growth system. Veggie surveys vary between pre-mission, in-mission and post-mission. Pre-mission administration occurred ~30 days before launch. The first in-mission administration occurred within three days of plant installation then transitioned to monthly administration during the crop growth experiment. Finally, the post-mission survey was completed within seven days after landing. The survey questions related to interacting with the Veggie chamber or the later Advanced Plant Habitat were originally collected using visual analog scales, with some changed to a 7-point Likert in later missions. The Veggie Survey items were directly applicable to the APH, requiring no changes between responses related to the crop growth systems. All data were rebinned to a 7-point scale to follow psychometric best practices prior to analyses. See full scales in Supplementary Information.

For the pre-mission Veggie Survey, crewmembers used the scale to respond to items about their general familiarity with plants and how they felt when tending to plants. Higher scores indicated a greater degree of that construct; for example, higher scores on enjoyment when tending to plants indicated more enjoyment. They also answered sensory items about their enjoyment when touching, smelling, seeing, and tasting plants.

For the in-mission Veggie Survey, items were carried over from the pre-mission survey but reframed to ask about crewmembers’ experiences related to interacting with the plants in space. Crewmembers also selected a specific survey version based on whether they interacted with the plant system(s) or not. If they did interact with the plants, they were asked to record minutes and enjoyment engaging in various crop growth activities (e.g., setup, watering, harvesting), rate various BHP outcomes (e.g., mood, autonomy, desire to consume), and describe any experiences with struggling plants. If a crewmember did not interact with the plants for a given survey occurrence, they were asked to complete the survey near the plant habitat with a shorter version of the survey that omitted the plant interaction questions.

For the post-mission Veggie Survey, crewmembers answered with a retrospective about their entire mission experience, including a prompt that asked about their experiences with struggling or dying plants.

### Study protocol

Crewmembers participating in ISS crop growth studies used the Veggie chamber (VEG; a unit open to the cabin atmosphere that was transparent to allow direct plant observation) or the Advanced Plant Habitat (APH or PH; a unit closed to the cabin atmosphere to allow for more environmental control, and with an opaque cover that could be removed to allow plant viewing). The Veggie chamber is the size of a two drawer filing cabinet with 0.13 meters squared growth area, while APH roughly double that size with a 0.2 meters squared growing area. Crewmembers installed and setup the crop growth experiment, then tended to the plants by watering, thinning the seedlings, removing debris (e.g., dead petals and leaves), pollinating, opening wicks, monitoring health and progress (via inspection, mass measurement, and photography), mitigating mold, and ultimately, harvesting. Crewmembers were required to wear gloves when handling plants and hardware to prevent produce contamination. Some crewmembers were able to consume plants grown on the ISS after produce was cleaned with sanitizing wipes. Hardware was cleaned after the final harvest of each growth experiment. Crewmembers were able to engage in voluntary viewing of the plants when desired. Note that some participants did not engage in all the crop growth experiment activities, depending on their mission timing in relation to the ongoing experiments.

The specific crop growth studies represented in this dataset include VEG-03 I-L, VEG-04 A-B, VEG-05, and PH-04, which saw several cultivars (i.e., multiple leafy greens, tomatoes, peppers). Several studies have addressed further information regarding these crop growth study designs and findings related to crop growth conditions (e.g., watering system testing, red- vs. blue-enriched light testing), botany, food safety, and nutrition^31^^,37–39^.

## Supplementary information

BHP Veggie 2025 - for npj mg SUBMIT -rev1.1 supplement

## Data Availability

The datasets generated and analyzed during the current study are available in the NASA Life Sciences Data Archive, https://nlsp.nasa.gov/.
