# Supplementary material for: Sustaining the Merry Space farmer with pick-and-eat crop production
Source: NPJ Microgravity. 2025 Oct 27;11:72. doi: 10.1038/s41526-025-00513-9 (PMC12559293; doi:10.1038/s41526-025-00513-9)
Supplement: Supplementary file 1 — BHP Veggie 2025 - for npj mg SUBMIT -rev1.1 supplement [file 41526_2025_513_MOESM1_ESM.pdf]

Supplementary Information

Supplementary Table 1. Behavioral Health and Performance Veggie Survey: Pre-Mission Demographics Survey

1.	Date of birth	MM/DD/YYYY		
2.	Biological sex	Male<br>Female		
3.	What is the highest level of education you have completed? (select one)	Less than High School<br>High School or GED<br>Some College<br>2-year College Degree (Associates)<br>4-year College Degree (BA, BS)<br>Master's Degree<br>Professional Degree (MD, JD, DDS, DVM, PsyD)<br>Doctorate (PhD, DSc, EdD, DFA)		
4.	What is your primary profession or field of study? (select one)	Arts and Entertainment<br>Biology<br>Business/Marketing/Accounting<br>Chemistry<br>Communications/Media<br>Computer Science/Technology<br>Culinary Arts<br>Education<br>Engineering<br>English Language and Literature<br>First Responder (police, fire, EMT)<br>Food Service<br>Foreign Language/Linguistics<br>Health Sciences/Medicine/Nursing<br>History<br>Hospitality/Tourism<br>Law<br>Mathematics/Statistics<br>Military/Armed Forces<br>Philosophy/Religion<br>Physics<br>Political Science/Government<br>Psychology - Research<br>Psychology - Clinical<br>Retail<br>Skilled Trade (construction, plumbing, artisan, etc.)<br>Sociology/Anthropology		
5.	How much time have you spent in your primary or field of study? If N/A, enter "0".	Years	Months	Weeks

Supplementary Table 1. Behavioral Health and Performance Veggie Survey: Pre-Mission Demographics Survey (continued)

6. How much total time have you spent living in space or space analog environments (e.g., ISS, HERA, NEEMO, MDRS, HI-SEAS?)? If N/A, enter "0".	Years	Months	Weeks
7. Before this current mission, how much total time have you spent living in confined or isolated operational environments (e.g. submarine, polar station, aircraft carrier, deep sea diving chamber, forward operating base, oil rig)? If N/A, enter "0".	Years	Months	Weeks
	**Left Anchor**	**Middle Anchor**	**Right Anchor**
8. Overall, how much team work do you engage in as part of your primary profession?	All individual work, rarely interact with others	Equal amount of individual and interactive /team work	All interactive /team work, rarely individual work

Supplementary Table 2: Behavioral Health and Performance Veggie Survey: Pre-Mission Survey

	**Left Anchor**	**Right Anchor**
**Familiarity**		
9. I am __________ with the work it takes to grow plants.	Totally Unfamiliar	Very Familiar
10. I __________ tended to plant life as a child.	Never	Always
11. As an adult, I __________ tend to plants.	Never	Always
**Tending to Plants**		
12. Tending to plants is _____.	Very Unpleasant	Very Enjoyable
13. Tending to plants is _____.	Tedious	Engaging
14. Tending to plants is _____.	Totally Meaningless	Very Meaningful
**Sensory Stimulation**<br>Instructions: In thinking about plants (e.g., plants, flowers, herbs, vegetables, fruits), please rate your level of enjoyment with the following:		
15. Looking at plants	Very Unpleasant	Very Enjoyable
16. Touching plants	Very Unpleasant	Very Enjoyable
17. Smelling plants	Very Unpleasant	Very Enjoyable
18. Tasting plants	Very Unpleasant	Very Enjoyable

Supplementary Table 3: Behavioral Health and Performance Veggie Survey: In-Mission Survey

**Veggie Activities**<br>Instructions**:** Throughout the time since your last Veggie Survey, please enter how many minutes you spent interacting with Veggie, then rate your enjoyment of each activity. For any given activity, if you did not interact with Veggie, the enter 0 minutes and skip the enjoyment rating.			
**Veggie Activity**	**Minutes**	**Left Anchor**	**Right Anchor**
1. Setup (hardware assembly, plant pillows, etc.)		Very Unpleasant	Very Enjoyable
2. Watering		Very Unpleasant	Very Enjoyable
3. Plant thinning		Very Unpleasant	Very Enjoyable
4. Debris removal		Very Unpleasant	Very Enjoyable
5. Pollinating		Very Unpleasant	Very Enjoyable
6. Wick opening		Very Unpleasant	Very Enjoyable
7. Photography		Very Unpleasant	Very Enjoyable
8. Harvesting		Very Unpleasant	Very Enjoyable
9. Consumption		Very Unpleasant	Very Enjoyable
10. Cleanup		Very Unpleasant	Very Enjoyable
11. Voluntary viewing		Very Unpleasant	Very Enjoyable
12. Other: [free response]		Very Unpleasant	Very Enjoyable

**Interacting with Veggie**<br>Instructions**:** Throughout the time since your last Veggie Survey, interacting with Veggie…		
	**Left Anchor**	**Right Anchor**
13. was ____.	Very Boring	Very Engaging
14. was ____.	Very Easy	Very Demanding
15. was ____.	Totally Meaningless	Very Meaningful
16. ____ my mood.	Diminished	Enhanced
17. ____ my performance of mission tasks.	Diminished	Enhanced
18. ____ my wellbeing.	Diminished	Enhanced
19. ____ my relationship with other crewmembers.	Diminished	Enhanced
20. ____my connection Earth.	Diminished	Enhanced
21. ____ my desire to harvest the plants.	Diminished	Enhanced
22. ____ my desire to consume the plants.	Diminished	Enhanced
23. ____ my consumption of food in general.	Diminished	Enhanced
24. In a typical week on orbit, what food(s) do you crave? Please list all food, not just plant-based. [free response]		
25. In a typical week on orbit, how many servings of fresh fruit and vegetables do you consume (think of one serving as an apple)?<br>a. 1-5<br>b. 6-10<br>c. 11-15<br>d. 16-20<br>e. More than 20		

Supplementary Table 3: Behavioral Health and Performance Veggie Survey: In-Mission Survey (continued)

**Sensory Stimulation**<br>Instructions: How effective is Veggie as a source of sensory stimulation for…?		
	**Left Anchor**	**Right Anchor**
19. Sight	Very Unpleasant	Very Enjoyable
20. Touch	Very Unpleasant	Very Enjoyable
21. Smell	Very Unpleasant	Very Enjoyable
22. Taste	Very Unpleasant	Very Enjoyable
**Struggling Plants**		
23. If any of the plants struggled or died, please describe this experience.<br>[free response]		
**Veggie General**		
24. Please feel free to share any additional thoughts or insights on interacting with Veggie.<br>[free response]		
